# The Emerging Role of Microglia in Neuromyelitis Optica

**DOI:** 10.3389/fimmu.2021.616301

**Published:** 2021-02-19

**Authors:** Tingjun Chen, Dale B. Bosco, Yanlu Ying, Dai-Shi Tian, Long-Jun Wu

**Affiliations:** ^1^ Department of Neurology, Mayo Clinic, Rochester, MN, United States; ^2^ Department of Neurology, Tongji Medical College, Huazhong University of Science and Technology, Wuhan, China; ^3^ Department of Neuroscience, Mayo Clinic, Jacksonville, FL, United States; ^4^ Department of Immunology, Mayo Clinic, Rochester, MN, United States

**Keywords:** microglia, neuromyelitis optica, complement C3, aquaporin-4, astrocyte-microglia communication, autoimmune, C3a receptor

## Abstract

Neuromyelitis optica (NMO) is an autoantibody-triggered neuro-inflammatory disease which preferentially attacks the spinal cord and optic nerve. Its defining autoantibody is specific for the water channel protein, aquaporin‐4 (AQP4), which primarily is localized at the end-feet of astrocytes. Histopathology studies of early NMO lesions demonstrated prominent activation of microglia, the resident immune sentinels of the central nervous system (CNS). Significant microglial reactivity is also observed in NMO animal models induced by introducing AQP4-IgG into the CNS. Here we review the potential roles for microglial activation in human NMO patients as well as different animal models of NMO. We will focus primarily on the molecular mechanisms underlying microglial function and microglia-astrocyte interaction in NMO pathogenesis. Understanding the role of microglia in NMO pathology may yield novel therapeutic approaches for this disease.

## Introduction

Neuromyelitis optica (NMO) is an autoimmune inflammatory disorder of the central nervous system (CNS) which mainly affects optic nerves and the spinal cord (1). Attacks upon optic nerves can often lead to blindness, while the extensive transverse myelitis induced within the spinal cord can cause weakness or paralysis in the legs and arms, loss of sensation, bladder and bowel function issues, and respiratory failure (2). Additionally, NMO disproportionately affects women and non-Caucasian populations and is a relapsing-remitting disease. Repeated attacks can lead to severe cumulative damage to optic nerves and the spinal cord (3). Therefore, prevention of new attacks is critical to mitigating long-term effects.

NMO was largely considered a subtype of multiple sclerosis (MS), until 2004 when Lennon and colleagues identified that autoantibodies directed towards aquaporin‐4 (AQP4-IgG, also called NMO-IgG) were a biomarker for NMO pathology (4). This key finding was used to distinguish NMO from MS and other inflammatory CNS demyelinating disorders. AQP4-IgG binds the ectodomain of AQP4 water channels that are located on the astrocytic end-feet embracing capillaries, ventricular walls, and pial-glial interfaces (4). AQP4-IgG can also sometimes be detected in a patient’s cerebrospinal fluid (CSF) (5). AQP4-IgG concentrations in the blood and CSF have been reported to correlate with clinical outcome (6–10). It should also be noted that antibodies against myelin oligodendrocyte glycoprotein (MOG-IgG) have also been suggested to induce NMO pathology (11). The idea that NMO pathology is related to pathogenic autoantibodies has lead plasma exchange and B cell depletion to be accepted as first line therapies for the acute phase of NMO (12).

The histopathology of NMO lesions also show prominent microglial reactivity in AQP4-rich CNS regions (13, 14). However, mechanisms underlying microglial activation in NMO remains largely unknown. It would be reasonable to believe that microglia are the first responders to signals emanating from astrocytes following their activation by AQP4-IgG. As such, this review focuses on the emerging evidence that microglia play an important role in AQP4-IgG related NMO pathogenesis.

## Histopathology and Microglial Reactivity in NMO Patients

The key hallmark of NMO immunopathology is the striking focal loss of AQP4 protein from astrocytes. Changes to glial fibrillary acidic protein (GFAP) expression in astrocytes varies in different brain regions and disease stages (14). In early lesions, astrocytes are spared and exhibit an activated phenotype (15). In later stages, when deposits of the complement terminal membrane attack complex C5b-C9 are abundant, terminal deoxynucleotidyl transferase dUTP nick end labeling (TUNEL) staining indicates astrocyte apoptosis and GFAP immunoreactivity is reduced (13–16). In some of these later lesions, microglia/macrophages contain GFAP remnants, indicating phagocytosis of astrocytes (14). Demyelination in NMO pathology, as shown by fast blue staining (14), generally accompanies astrocytic pathology (17) and local inflammation (18).

*In vitro* studies of NMO pathogenesis found that the binding of AQP4-IgG to its antigen induces AQP4 internalization (19, 20), reducing water permeability and spurring astrocytes to release various cytokines and chemokines (21). Coincidently, when AQP4 is internalized, excitatory amino acid transporter 2 (EAAT2) also disappears from astrocyte surface membranes (20). In the presence of requisite complement components, astrocytes undergo cytolysis during an NMO attack (13, 19, 22). Additionally, myelin loss in both grey and white matter is an immunohistochemical characteristic of fully established NMO lesions (14). However, demyelination appears to be a secondary event following the interaction of AQP4-IgG with astrocytes (17) and promotion of local inflammation (18). Neuronal loss has also been reported in the cerebral cortex of NMO patients, possibly explaining the cognitive impairment that is sometimes observed (23). As for MOG-IgG positive NMO patients, myelin loss was significant but AQP4 was preserved and dystrophic astrocytes were absent (24). Although MOG-IgG frequently co-existed with anti-N-methyl-D-aspartate (NMDA) receptor IgG, the prognosis in these patients are usually better than AQP4-IgG positive patients, indicating differences in pathogenesis (25).

In NMO patients’ CNS, perivascular regions contain accumulations of lymphocytes, neutrophils and eosinophils (14–16). The abundance of these cellular infiltrates suggests disruption of the neurovascular unit following interaction of NMO-IgG with AQP4 in the vicinity of the blood-brain-barrier (BBB) (13). This is not surprising, considering astrocyte end-feet are an integral part of the neurovasculature. Consistently, AQP4 loss in the choroid plexus coinciding with C9 neoantigen (C9neo) immunoreactivity on choroidal epithelial membranes is evidence of focal BBB pathology (13). C9neo deposition can also be detected in MOG-IgG positive patients, but on myelin sheaths (24). Although peripherally activated B cells can enter the parenchyma through intact BBB (26), loss of astrocytic end-feet may further facilitate entry of either AQP4-IgG or AQP4-IgG secreting B cells into the CNS (27).

In addition to loss of AQP4 and astrocytes, neuronal injury, demyelination, microglial activation, and macrophage infiltration are prominent in AQP4-IgG seropositive NMO pathology (14). Microglia and macrophage reactivity is indicated by both morphological criteria (*i.e.*, amoeboid morphology) and positivity for the lysosome marker CD68 (14, 16). However, conventional histopathology cannot distinguish macrophages (infiltrating monocytes) from resident microglia in the CNS. Although there are conflicting reports as to whether the number of monocytes within circulation changes (28, 29) in response to NMO-IgG, infiltration of monocytes into the CSF is believed to happen (30). Indeed, there is an increased number of microglia-like cells within human NMO pathology. However, it is difficult to determine whether this is due to local microglial proliferation or monocyte infiltration after NMO-IgG attacks.

It should also be noted that reported CNS tissue distribution of activated microglia/macrophages is different between NMO and MS. In MS, microglial activation is wide-spread in the meninges and subpial cortex (31), while in AQP4-IgG positive NMO it is largely confined to regions that normally are AQP4-rich, like the CSF–brain interface in the corpus callosum, hypothalamic, and periventricular areas (13, 14, 32). Conversely, in MOG-IgG positive NMO, microglia/macrophages also appear in subpial, which is similar to MS; but it is associated with CD4 inflammatory infiltration and not CD8 T cells, which are abundant in MS (24). Additionally, microglia/macrophage reactivity in NMO lesions are surrounded by evidence of complement activation, which is an infrequent finding in MS lesions (14). Consistent with the role of complement in microglial function, microglia are able to secret complement components to activate astrocytes and mediate neuronal injury in a variety of neurological disorders (33–35). These complement components can also further activate microglia (36–38). Therefore, the difference in complement-related microglia activation patterns between MS and NMO indicate divergent molecular mechanisms. Nevertheless, clinical histopathology results strongly suggest the involvement of microglia in NMO pathogenesis. As such, NMO animal models are needed to investigate the precise function and mechanism of microglia in NMO.

## Rodent Models of NMO

To understand the cellular and molecular mechanisms underlying the pathogenesis of NMO, several rodent models of NMO have been developed. Since AQP4-IgG was the original pathological cause of NMO, animal models that employed AQP4-IgG to develop pathology are the most common. As such, we will focus predominantly on AQP4-IgG related animal models.

Initial animal models were derived from experimental autoimmune encephalomyelitis (EAE) where pathology was induced *via* adoptive transfer of AQP4-IgG into animals with preexisting myelin targeted encephalitogenic T cells (5, 39–43). However, these models did not display any of the immunology characteristics of NMO. In particular, there were no AQP4 specific immune reactions (44–47). Another early model involved transplantation of AQP4-specific T cells into naïve animals (48–53). However, this model also did not induce the characteristic AQP4 loss unless AQP4-IgG were co-injected (48, 49). More recently, new models that more closely mimic the clinical features of AQP4-IgG related NMO have been developed (**Table 1**).

**Table 1:** AQP4-IgG related NMO animal models.

NMO model	Animals	Site of pathology	AQP4 loss	GFAP loss	Complement activity	Microglia activity	Demyelination	Functional impairment	Reference
AQP4-IgG seropositive	mouse	Peripheral Organs	No	No	No	No	No	No	(54)
	rat	Brain (needle)	Yes	Yes	Yes	Yes	Yes	Yes	(55)
		Brain (Ultrasound)	Yes	Yes	Yes	Yes	Yes	Unclear	(56)
		Brain/Spinal cord/Optic nerve							(57)
AQP4-IgG with human complement	mouse	Brain/Spinal cord	Yes	Yes	Yes	Yes	Yes	Unclear	(58)
		Brain						Yes	(59, 60)
		Optic nerve						Unclear	(55)
		Spinal cord						Yes	(61)
AQP4-IgG without human complement	rat	Brain	Yes	Yes	Yes	Yes	Yes	Unclear	(62)
		Brain			Yes	Yes	Yes	Yes	(63)
		Brain			No	No	No	Yes	(64)
		Optic nerve/retina			Yes	Yes	Yes	Yes	(65, 66)
		Spinal cord							(67, 68)
	mouse	Spinal cord	Yes	Yes	No	Yes	Yes	Yes	(69)
				No	C1q and C3				(70)

### AQP4-IgG Seropositive Models

Some recent rodent models of NMO have utilized passive transfer of AQP4-IgG into animals to induce clinically relevant pathology and provide early evidence of microglial activation in NMO pathology (58, 61, 71). However, one complication in using passive transfer is that acute intravenous injection of AQP4-IgG has difficulty producing neurology dysfunction or NMO pathology (54, 55), as AQP4-IgGs cannot pass through an undamaged BBB. When the BBB is physically disrupted by needle puncture or ultrasound, NMO-liked pathology can be induced near leakage sites (54, 56). Repeated intraperitoneal AQP4-IgG injections has also been used to successfully induce wild-spread NMO-liked pathology in rats (57). The neurological abnormalities displayed in this model included neuropathic pain, salivation (correlates to nausea/vomiting behavior in rats/mice) (72), problems with balance, and motor impairment (57).

AQP4-IgG in seropositive models entered the CNS *via* three routes: (1) Circumventricular organs, which are highly vascularized structures surrounding the third and fourth ventricles and characterized by a lack of BBB (73). Using this route AQP4-IgG induces a lesion with AQP4 loss but intact GFAP staining. However, in circumventricular organs, microglia were not activated after repeated intraperitoneal AQP4-IgG injection. (2) Meningeal vessels and veins of the Virchow Robin spaces. Using this route AQP4-IgG induces meningitis 120 h after the initial intraperitoneal injection of AQP4-IgG. T cell, neutrophil and microglia activity were found in subpial lesions. Notably, microglial activity was accompanied with astrocyte damage in these areas. (3) Finally, parenchymal vessel unrelated to the meninges. Perivascular lesion deep within the parenchyma showed significant AQP4 loss and large number of activated microglia. In some lesions, strong complement deposition coincided with the loss of astrocytes and activation of microglia, which is similar with lesions of NMO medullas in patients (15).

### Direct AQP4-IgG Injection Together With Human Complement

In these animal models, either AQP4-IgG derived from NMO patients or recombinant monoclonal AQP4-IgG is administered directly into the rodent CNS together with human complement (59, 60, 62). In wild type mice, when patient-derived AQP4-IgGs were intracerebrally injected acutely with human complement, matured NMO pathology including AQP4 and GFAP loss, myelin damage, complement deposition, and axon injury was observed (59). CD45^+^ microglia/macrophages were also widely observed in the ipsilateral brain. Moreover, animals were more likely to turn to the injection side during Y maze test, indicating neurological dysfunction in injected hemisphere of brain. In contrast, mice treated with human control IgGs and complement exhibit no behavior abnormalities, AQP4 loss, astrocyte injury, axon loss, or microglia/macrophages activation, suggesting that APQ4-IgG is the main trigger for pathology (59).

Chronic infusion of recombined AQP4-IgG with human complement was also shown to induced astrocyte damage, neutrophil infiltration, eosinophil infiltration, and microglia/macrophage activity in mice (58, 60). Additionally, deficiencies in complement regulator CD59 expression significantly increased the pathology induced by AQP4-IgG, including AQP4 loss, complement deposition, and CD45^+^ cell activity (55, 61, 64). These results are quite surprising, as complement proteins themselves are known to induce microglial activity (74–76) followed by neuronal damage (77) and demyelination (78). Therefore, further studies are needed to clearly delineate the respective contribution of AQP4-IgG and complement to the outcomes of NMO-like phenotypes in these models.

### Direct AQP4-IgG Injection Without Human Complement

Co-injection of complement, however, may not be necessary to induce pathology. In rodents, AQP4-IgG injection without human complement was able to induce neurological dysfunction and NMO pathology within the brain, retina, and optic nerves (62, 63, 65–68, 70). Microglial reactivity was observed within areas of AQP4 loss (64, 68–70). Administration of clodronate liposomes, which efficiently depletes microglia/macrophages, significantly reduced lesion size, suggesting that microglia/macrophages contributed to NMO pathology (62).

Nevertheless, there is still debate as to whether complement has a role in these types of models. For example, in CD59 deficient mice, AQP4-IgG induced worse functional impairment, more NMO-like pathology, and stronger microglial activity (67). These results indicate that AQP4-IgG could induce endogenous complement expression, which in turn facilitates the toxicity of AQP4-IgG. However, in other reports, no complement terminal protein was detected (64, 68, 70). Additionally, pretreatment with cobra venom factor, which inhibits complement activity (79), did not rescue functional impairment and pathology in the retina (65). Therefore, it is unclear whether the pathology and neuronal dysfunction induced in these models truly involved complement related mechanisms.

## Microglial Activation in Established Animal Models of NMO

As mentioned above, microglial activation has been observed in most animal models of NMO. However, it is unknown whether microglial activation is the cause or consequence of NMO pathogenesis. Moreover, it is unclear as to whether complement is involved in microglial activation, considering microglia reactivity has been observed in both complement dependent (59, 61, 67) and independent models (65, 69, 80). Serum concentrations of complement have also varied across different NMO cases, making it difficult to ascertain whether complement toxicity is initiated by AQP4-IgG (81–84). Nevertheless, the clear early events in NMO are astrocytic reactivity after AQP4-IgG binding (20) and AQP4 loss (22). AQP4-IgG activated astrocytes may then produce cytokines, which induce local inflammation and microglial activation (19–21, 70). Microglia, as the resident CNS immune cell, should typically be the first responders to reactive astrocytes following initiation of NMO pathology. However, limited direct evidence exists that illustrates microglial function and activation mechanisms in NMO. As such, a new murine model of NMO has recently been developed to determine where microglia fit into NMO pathology (70).

## A New Murine Model of NMO That Employs Chronic AQP4-IgG Intrathecal Infusion Reveals the Important Role of Microglia

A new mouse model of NMO has been established that utilizes continuous infusing of AQP4-specific IgG into the spinal subarachnoid space, without exogenous complement (70). Recipients of monoclonal AQP4-IgG or NMO-patient-derived IgG displayed progressive motor impairment and immunohistopathology compatible to early clinical NMO pathology. The pathology showed that AQP4 levels in spinal cord tissue were decreased after AQP4-IgG infusion, but GFAP immunoreactivity was increased. Therefore, AQP4 is likely internalized but astrocytes are not undergoing apoptosis, mimicking the early stages of NMO. In AQP4 null mice, AQP4-IgG did not induce motor impairment, confirming that autoantibodies targeting AQP4 are responsible for triggering the pathology. Additionally, complement terminal proteins were not detected in this model. Instead, it was demonstrates that microglia play a central role in the observed pathology (70).

### Microglia Are Required for NMO Pathogenesis

Activation of microglia/macrophages as determined by Iba1, CD11b, CD68, and C1q staining was significantly increased within the AQP4 loss lesion (70). These results are consistent with microglial/macrophages activation in human NMO patients (13, 14, 16). Interestingly, in AQP4^-/-^ mice, AQP4-IgG did not activate microglia/macrophages, indicating that AQP4-IgG activated microglia *via* astrocyte signaling. Recent studies have proposed that microglia induce reactive astrocytes in many disease contexts, such as Alzheimer’s disease, Huntington’s disease, Parkinson’s disease, amyotrophic lateral sclerosis, and MS (35, 85). The results in the mouse model of NMO demonstrated that astrocytes can induce the activation of microglial/macrophages. Astrocytic modulation of microglia is also observed in development and diseases (86, 87). For example, astrocytes release transforming growth factor (TGF)-β (88, 89) or interleukin 33 (IL-33) (86, 90) to promote microglial pruning of synapses in development. Under disease conditions, astrocyte up-regulate lipocalin-2 (LCN-2) (91) or orosomucoid (ORM) (92) to activate microglia, which leads to neuro-inflammation and degeneration.

To further investigate this proposed relationship between astrocyte-microglia communication and NMO pathology, microglia ablation approaches were employed (70). When microglia were depleted, AQP4-IgG infusion did not induce motor impairment. More strikingly, when microglial replenishment started around day 5 after ablation, motor dysfunction appeared (70), suggesting that microglia are critical mediators of the behavioral impairment. It is important to point out that microglia ablation did not prevent AQP4 internalization or astrocytic activation induced by AQP-IgG infusion. This is consistent with a previous *in vitro* study, which demonstrated that autoantibody-induced AQP4 internalization was microglia independent (20). Among rodent models with clinical comparable NMO pathology (40, 55, 61, 69, 70), this new study provides the direct evidence of microglia in motor dysfunction. The microgliogenic pathology separates NMO from other neuro-inflammatory diseases like MS. For example, in MOG induced experimental autoimmune encephalomyelitis (EAE) models, during disease onset, infiltrated macrophages induce inflammation (45), while microglia clear debris and limit inflammation (46). Additionally, in demyelinating diseases, microglia trigger remyelination and promote recovery (46, 93–95). Therefore, although the study suggests a damaging role for microglia in the early phases of NMO pathogenesis, microglia function could be more diverse at later phases of NMO.

### Astrocyte-Microglia Interaction Is Mediated by C3-C3aR Signaling

**Figure 1:**
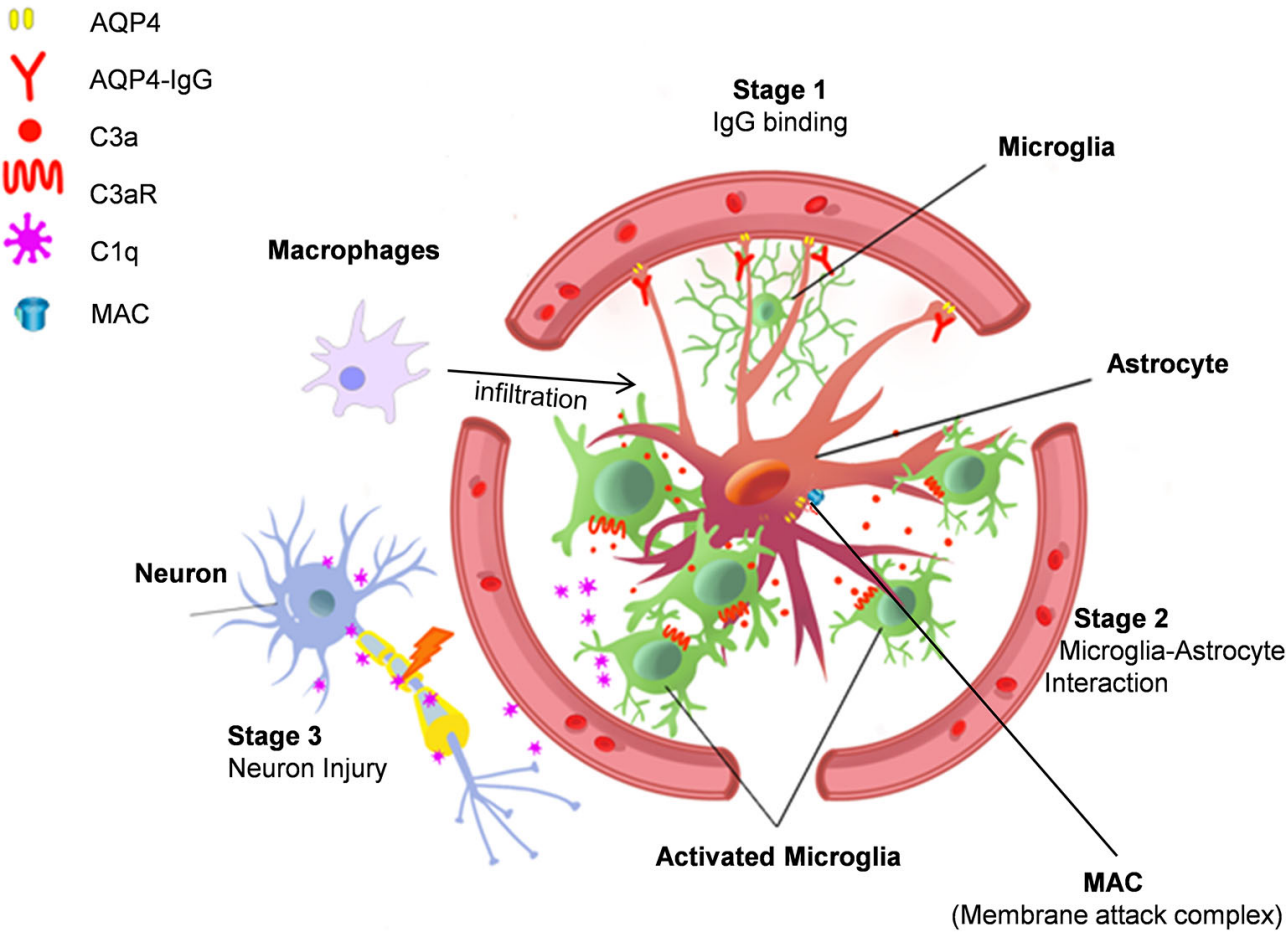
Astrocyte-microglia interaction drives NMO pathology. Stage 1. AQP4-IgG binds to AQP4 water channel protein in astrocyte end-feet and induces AQP4 protein internalization. Stage 2. AQP4 internalization activates astrocytes, which then increases complement C3 expression to induce microglia chemotaxis. Stage 3. Activated microglia interact with astrocytes and release C1q, which may cause neuronal injury. Additional mechanisms in NMO pathogenesis include complement dependent cytotoxicity mediated by membrane attack complexes (MAC) and focal inflammation induced by macrophage infiltration.

After AQP4-IgG infusion, microglia and astrocyte cell somas were found to overlap after AQP4-IgG infusion (70). This observation suggests astrocyte-microglia physical interaction in this animal model of NMO. While microglial process convergence towards neurons has been previously reported (96, 97) this is the first time that similar events have been observed with astrocytes after AQP4-IgG infusion. Complement C3 is typically absent in astrocytes under physiological conditions, but strongly expressed under pathological conditions (35). Consistently, AQP4-IgG infusion was found to upregulate astrocytic C3. AQP4-IgG infusion also did not induce motor impairment in C3^-/-^ animals. Similar to ATP (98, 99), the cleavage produce of C3, C3a, can act as a chemoattractant, possibly inducing the observed microglial processes convergence through C3a receptor (C3aR) expressed in microglia (100). In C3^-/-^ and C3aR^-/-^ mice after chronic infusion of AQP4-IgG, even though AQP4 loss still occurred, astrocyte-microglia interaction and motor deficits were largely abolished (70). Therefore, astrocyte-microglia interaction is likely mediated by C3-C3aR signaling and this interaction is a driver of this model’s NMO pathogenesis (**Figure 1**).

Complement C3 shapes brain function in healthy and diseased conditions (34, 101–103). Recent experimental evidence indicates that microglia are critical for C3-C3aR signaling in neurodegenerative diseases (104, 105). In addition, C3 signaling can also participate in neurodegeneration by activating microglial complement receptor 3 (CR3) (34, 103, 106, 107). Indeed, exogenous human complement C3 can activate murine microglia (105). Administration of AQP4-IgG with complement induces astrocytes depletion in hours (108), which skipped the initial stage of astrocytic reactions in NMO pathology (15, 19, 20). Astrocyte acute ablation likely results in neuronal damage (109), but the question is whether such models really reflect the unique pathology of NMO. In animal models with AQP4-IgG chronic infusion without complement, activation of astrocytes, microglia, and neuronal damage happen in a temporally and spatially dependent manner (68, 70). The downstream cascade might also involve microglial phagocytosis of C3 labeled neurons/synapse, which is observed during neuronal development, neuro-inflammation, and neurodegeneration diseases (35, 103, 106).

### Neuronal Damage Is Possibly Mediated by Microglia-Derived C1q

After AQP4-IgG chronic infusion, microglial C3aR induces the expression of complement C1q in microglia (70), the complement cascade initiator (110). However, functional impairment does not seem related to complement induced toxicity for two reasons. First, there was no evidence of terminal complement complexes in this model. Second, astrocytes were activated but not injured. Therefore, it is possible that microglial C1q induces cell stress *via* different mechanisms other than classical complement cascades. Notably, C1q is able to inhibit mitochondrial function (111), which can lead to myelin and neuronal injury (107, 111, 112). Additionally, C1q can directly bind synapses and induce neuronal damage in a microglial phagocytosis dependent manner (106, 113). However, further study is needed to test whether there is complement-dependent microglial “phagoptosis” of neurons in NMO. Regardless, this model of NMO demonstrates a clear role for microglia in which microglial C1q may induce neuronal dysfunction in a complement cascade independent manner (**Figure 1**).

## Targeting Microglia for NMO Treatment

Based on our recent understanding of critical microglial function in NMO, we propose that microglia may represent a novel therapeutic target for NMO treatment. Typically, acute NMO attacks are first treated with intravenous methylprednisolone for 3~5 days (11), combined with plasma exchange (PLEX) or immunoabsorption (114) to help reduce AQP4-IgG concentrations (115). Subsequently, low doses of prednisone/prednisolone are used for preventative immunosuppressive therapy, however this can induce serious side effects (116). Additional therapeutic treatments for NMO include Azathioprine and Rituximab, which inhibit lymphocyte and CD19^+^ B cell activity (117). Mycophenolate mofetil has also been useful to inhibit B cells and T cells, but patients will have elevated risk of leucopenia when combined with other immunosuppress therapies (118). It remains to be determined whether these immunosuppressive therapies reduce microglial activation when they exert their therapeutic effects.

The most exciting breakthrough in NMO therapy is eculizumab, which greatly reduces the risk of relapse in AQP4-IgG seropositive patients (3). Eculizumab neutralizes complement C5, which is mainly expressed by microglia in the CNS (74). Interestingly, C5a receptors are highly and exclusively expressed by microglia (119). Clinical evidence indicates NMO pathology is mediated by immunoglobulin and terminal complement complex (C5b-9; C9neo) (16), highlighted the importance of complement dependent cytotoxicity (CDC) in NMO. Besides immunoglobulin mediated astrocyte CDC, the demyelination in NMO lesions can be induced by production of anaphylatoxins (C3a, C4a and C5a) and opsonins (C3b and C1q) that recruit inflammatory cells (120), enhance antibody-dependent cell-mediated cytotoxicity (ADCC) (121), and facilitate phagocytosis (120). Although microglia reactivity has not been examined in eculizumab treated patients yet, early studies already implied how complement C5 inhibition modulate microglia function in CNS disease. First, inhibition of C5a receptors reduced microglia activity and provided strong benefit in mouse models of Alzheimer’s disease (112). Moreover, microglial C5a is a strong chemoattractant for polymorph nuclear cells (122), mediates eosinophil and neutrophil degranulation (123, 124). Therefore, future studies are needed to investigate whether eculizumab can reduce the relapsing risk of NMO *via* inhibition of microglial C5 receptor signaling.

Lastly, other microglia-targeting therapeutic approaches could be considered for NMO treatment. First, microglial inhibitor minocycline has been shown to reduce microglial activation in many CNS disorders (125–127). Clinical trials of minocycline are currently in progress for schizophrenia, autism, anxiety, and bipolar disorders (128, 129). Second, colony stimulating factor 1 (CSF1) receptor antagonists, such as PLX3397 or PLX5562, can deplete microglia *via* oral administration (130). PLX3397 (pexidartinib) was approved by FDA for treatment of giant-cell tumor of the tendon sheath in 2019 (131). Whether PLX compounds can be used for NMO therapy still need further investigation. However, microglia depletion/inhibition *via* CSF1 receptor antagonists may also result in side effects including affecting hematopoiesis and the function of macrophages in mice (132). Last but not least, C3 signaling such as C3 and C3a receptor, is critical for microglial activation and motor impairment in NMO (70). Inhibition of C3aR or neutralization of C3a may provide therapeutic effects. The C3a receptor is an especially attractive target as it is highly expressed in activated microglia (78, 104, 105).

## Future Directions and Conclusion

Recent studies have demonstrated the emerging role of microglia in NMO (70). Targeting microglia for NMO treatment is promising but still challenging, considering that microglia share many common molecules with peripheral immune systems (133, 134). To develop microglia specific treatments, future studies are needed to further our understanding of the molecular mechanisms underlying the role of microglia in NMO. Additional considerations include accounting sex differences in NMO, where the female to male ratio in NMO patients is above 8:2 (135). Coincidently, microglial function show clear sex differences evidenced by studies in chronic pain (136, 137), depression (138), stroke (139), and aging (140). It would be interesting to test whether female microglia may have stronger complement or proinflammatory activation in response to AQP4-IgG. In addition, the regional heterogeneity of microglia function is emerging. Whether microglial heterogeneity contributes to preferential development of lesions in optic nerves and the spinal cord warrants further investigations. Finally, it is unknown what the role of microglia is during the remission phase of NMO. Current animal models demonstrate that motor dysfunction can partially recover after AQP4-IgG infusion stopped (68, 70). Thus, the question remains as to whether microglia initiates NMO remission due to microglial reparative function or releasing trophic factors.

In a variety of neurological disorders (*e.g.*, stroke, chronic pain, epilepsy, and neurodegenerative diseases), microglia play beneficial or detrimental function depending on the context and timing of their activation (141–145). The novel mouse model of NMO presented in this review demonstrates a critical role for microglia in the evolution of motor impairment and the neuropathology progression initiated by AQP4-IgG activated astrocytes (70). With the emergence of new genetic tools for microglial research (146, 147), the function and mechanisms of microglia in NMO will be revealed. These will corroborate the idea that microglia may represent a novel therapeutic target in NMO treatment.

## Author Contributions

TC and L-JW conceived the study and wrote the manuscript. DB, YY, D-ST, and L-JW edited the manuscript. All authors contributed to the article and approved the submitted version.

## Funding

The work is supported by the National Institutes of Health (R01NS110949, R01NS088627, R01NS112144, R01NS110825, R21AG064159) to L-JW.

## Conflict of Interest

The authors declare that the research was conducted in the absence of any commercial or financial relationships that could be construed as a potential conflict of interest.
